# Stimulant Medications and Bone Health

**DOI:** 10.1007/s11914-026-00965-2

**Published:** 2026-05-14

**Authors:** Christine M. Swanson, Julie H. Wolfe, Chadi A. Calarge

**Affiliations:** 1 Division of Endocrinology, Metabolism and Diabetes, University of Colorado Anschutz, 12801 E. 17th Ave. Mail Stop 8106, Aurora, CO 80045 USA; 2 Geriatric Research, Education and Clinical Center, Rocky Mountain Regional VA, Aurora, CO USA; 3 Department of Psychiatry, University of Colorado Anschutz, Aurora, CO USA; 4 Menninger Department of Psychiatry and Behavioral Sciences, Baylor College of Medicine, Houston, TX USA

**Keywords:** Bone mineral density (BMD), Fracture, Stimulant medication, Methylphenidate, Amphetamine, Attention deficit/hyperactivity disorder (ADHD)

## Abstract

**Purpose of Review:**

This review summarizes the potential impact of stimulant medications on bone health, to raise awareness and optimize their safe use.

**Recent Findings:**

Stimulant medications can impair bone health through inadequate nutrient/calorie intake and stimulation of the sympathetic nervous system, which regulates bone metabolism. However, their use decreases fracture risk in younger populations ($\:\le\:$25 years old) with attention deficit/hyperactivity disorder (ADHD), likely through reductions in impulsivity and injury. Special care may be needed when fractures occur while using these medications, as recent data suggest psychostimulants are associated with impaired fracture healing.

**Summary:**

Prescription and non-prescription use of psychostimulants have increased. Their ultimate effect on bone health may be a balance between effective behavioral modification and adverse effects on bone metabolism related to nutrition and sympathetic activation. Future research should investigate how psychostimulants affect acquisition of peak bone mass, stability of bone mineral density (BMD) when used through mid/late-life, and fracture healing.

## Introduction

Stimulant medications are the first-line treatment for attention deficit/hyperactivity disorder (ADHD) [1–3]. Methylphenidate and amphetamines inhibit the reuptake of dopamine and norepinephrine while amphetamines also increase dopamine release resulting in effective ADHD behavioral modification [1]. Some side effects of this enhanced sympathetic nervous system (SNS) signaling are well described (e.g., tachycardia). However, the medications’ effects on other SNS-responsive tissues, such as bone, are not well understood and may go unrecognized until later in life.

Psychostimulant utilization has increased, particularly in adults, driven by increases in both ADHD prescriptions and non-prescription use [1, 4–11]. Stimulants are among the most commonly misused medications. Up to 9% of school-aged children and $\:\le\:$35% of college-aged students report psychostimulant misuse [7–10]. Furthermore, a 2023 National Survey estimated 3.9 million people $\:\ge\:$12 years old misused psychostimulants [10]. This misuse, which is likely under-reported [8], was most prevalent in the 18–25 year age group [10], who are particularly vulnerable to skeletal insults as they near the end of the finite period of bone modeling.

Bone modeling, the process of growing and consolidating the skeleton during youth, culminates with the achievement of peak bone mass during the mid-20s [12]. Peak bone mass is a major determinant of skeletal strength and fracture risk later in life [12]. Therefore, skeletal insults during youth impact fracture risk acutely and in the latter decades of life. This finite period of bone anabolism overlaps with the peak ages of ADHD diagnosis [2] and treatment with psychostimulants. These medications can reversibly impair growth, due to appetite suppression and nutritional issues [2]. In addition, preclinical and clinical data have elucidated how the SNS regulates bone metabolism with excess sympathetic tone being detrimental to bone health [13]. Therefore, independent of their effects on nutrition, physical activity, weight, and linear growth, the excess SNS tone induced by psychostimulants could represent a novel risk factor for osteoporosis and fracture when used during the critical period of bone anabolism and even throughout adulthood. Although most data suggest psychostimulants actually decrease fracture risk in younger populations ($\:\le\:$25 years old) with ADHD, data on the skeletal risks of long-term use, particularly in adult and advanced age groups, are not well understood despite increasing prevalence.

This review will examine psychostimulant pharmacology, SNS-regulation of bone metabolism, and data on how these medications influence bone mineral density (BMD) and fracture risk in ADHD. Awareness about potential skeletal effects of these medications among prescribers, users, and their families is low [14]. Therefore, the goal of this review is to help raise awareness to optimize their safe use in patients that require them.

## Methods and Findings

Pubmed electronic database searches were conducted through October 2025 using the terms “stimulant medications” OR “methylphenidate” OR “amphetamine” AND “bone” OR “fracture” OR “bone mineral density” OR “BMD”. A total of 238 articles were identified and screened for relevance based primarily on title and, if needed, abstract. Only English language articles were included. Articles were excluded if published in web-based journals without rigorous peer-review (e.g., Cureus) or reported data in chronic illicit stimulant users due to confounding. Relevant citations were also identified in the references lists of articles from the above search and in the authors’ personal databases. A total of 80 articles published between 1995 and 2025 were included in this review.

## Attention Deficit/Hyperactivity Disorder (ADHD)

ADHD is an increasingly prevalent neurodevelopmental condition [2, 3, 15, 16]. In the U.S., it is estimated that > 10% of children and adolescents have ADHD [16, 17]. It is diagnosed clinically based on the characteristic symptoms of inattention, hyperactivity and impulsivity that impair learning and daily activities [2]. The peak age of diagnosis is 9.5 years old with ~ 92% of individuals diagnosed by 25 years old [2]. It often persists into adulthood [11], and although the prevalence of ADHD symptoms and diagnosis can decline with age [2, 18], a recent national survey found that 25% of adults suspect they have undiagnosed ADHD [19]. Comorbidities are prevalent (e.g., mood disorders, substance use) which can affect ADHD symptomatology and bone health, regardless of additional medication use (e.g., SSRIs, antipsychotics) [2]. 

In U.S. and most other ADHD guidelines, pharmacotherapy in the form of stimulant, or less commonly non-stimulant medication [1, 2, 6] (e.g., atomoxetine, clonidine, guanfacine, viloxazine), with or without behavioral therapy is considered first line treatment for patients $\:\ge\:$6 years old [2, 3]. Psychostimulants were first recognized to improve inattention, hyperactivity and impulsivity in 1937 [1, 20], and are used by the majority of children and adolescents with ADHD [17, 21]. Historically, stimulant medication use wanes after completion of formal education [22]. However, with increasing recognition of adult symptoms, psychostimulant initiation and continuation beyond adolescence is more prevalent [11].

### Stimulant Medications (Table 1 [1, 2, 6])

Methylphenidate/dexmethylphenidate and amphetamines (e.g., dextroamphetamine, lisdexamfetamine), are FDA-approved to treat ADHD (all), narcolepsy (methylphenidate, dextroamphetamine), and binge-eating disorder (lisdexamfetamine). The medications come in many formulations (Table 1). Administration routes vary when used for nonmedical purposes (e.g., snorting, smoking, injection) [23].

**Table 1:** Stimulant Medications FDA-Approved for ADHD

Generic (Trade) Name(s)	Formulation(s)
Amphetamines	
Amphetamine sulfate (Adzenys [ER, XR-ODT], Dyanavel XR, Evekeo [IR, IR-ODT])	Oral tablets (immediate or extended release), orally disintegrating tablets, or oral liquid
Dextroamphetamine/Amphetamine (Adderall [IR, XR], Mydayis)	Oral tablets, oral capsule (extended release)
Lisdexamfetamine (Vyvanse)	Oral capsule or chewable tablet
Methamphetamine hydrochloride (Desoxyn)	Oral tablet
Dextroamphetamine sulfate (Dexedrine, Zenzedi, ProCentra, Xelstrym)	Oral tablets (immediate or extended release), oral liquid, transdermal patch
**Methylphenidate**	
Methylphenidate (Ritalin [IR, SR, LA]), Concerta, Quillichew ER, Quillivant XR, Cotempla XR-ODT, Aptensio XR, Methylin [chewable, ER], Metadate [CD, ER], Jornay PM, Relexxii, Daytrana	Oral tablet (immediate or extended release), chewable tablet (immediate or extended release), oral liquid (immediate or extended release), capsule (extended release, controlled delivery, delayed release), transdermal patch, oral disintegrating tablet, osmotic-release oral system tablet, multilayer extended-release capsule
Dexmethylphenidate (Azstarys, Focalin [IR, XR])	Oral tablet (immediate or extended release), capsule (extended release)

*IR* = immediate release, *ER/XR* = extended release, *ODT* = orally disintegrating tablets, *SR* = sustained release, *LA* = long-acting, *CD* = controlled delivery

The most common adverse events of stimulant medications include appetite suppression with subsequent weight loss or impaired linear growth/weight gain, and sleep disturbance [1]. These tend to be dose-dependent. While appetite suppression is one of the most common side effects occurring in up to 50% of children [24], it is usually mild to moderate and most pronounced within 6 months of medication initiation or titration, resulting in ~ 1.4 kg loss of fat mass [25, 26]. In severe cases, it can lead to nutritional deficiencies, including calcium and vitamin D, which may limit attainment of peak bone mass [14]. Less common adverse events include increases in heart rate and/or blood pressure, abnormal movements, seizures and psychosis [1, 27]. Despite increased use of stimulant medications initiated during childhood/adolescence into and throughout adulthood, data on the risks of long-term use, particularly in adult and advanced age groups, are not well understood [5, 11]. While this review focuses on stimulant medications used primarily for ADHD, the discussion may apply to other medications with similar mechanisms of action (e.g., modafinil).

### SNS Regulation of Bone Metabolism

Bones are innervated by sympathetic nerves [28] and sympathetic tone is an important regulator of bone metabolism [13, 29–31]. By inducing catecholamine release and/or blocking their reuptake, stimulant medications activate alpha and beta ($\:\beta\:$)-adrenergic receptors, thereby potentially impacting bone health

Khosla and colleagues elegantly demonstrated the role of adrenergic signaling in the regulation of bone metabolism in humans [13]. First, using bone biopsies in young and older women, they demonstrated that adrenergic receptors (β_1_ and β_2_, not β_3_) are expressed in human bone tissue, specifically osteoblasts. Second, they showed that treatment with selective β_1_-blockers was associated with better bone microarchitecture than nonusers. Third, in a randomized placebo-controlled trial, postmenopausal women who received atenolol or nebivolol (i.e., highly selective β_1_-blockers), but not propranolol (nonselective β-blocker), had reduced levels of C-telopeptide of Type I collagen (CTX), a marker of bone resorption, and increased BMD at the radius relative to placebo. These three independent lines of investigation (i.e., biological plausibility, epidemiological associations, interventional clinical trial data) provided strong evidence for how excess adrenergic signaling, specifically mediated by the β_1_-receptor, is detrimental to bone health and how blocking it may have skeletal benefits in humans [13].

Other investigators have also demonstrated the role of sympathetic tone in the regulation of bone remodeling: [29–31]

- Sympathetic activity uniquely uncouples the process of bone turnover [28, 32]. Through direct inhibitory effects on β_2_-receptors on osteoblasts and subsequently increased RANKL expression by osteoblasts to stimulate osteoclasts [13, 28, 33, 34], leptin-dependent sympathetic activity suppresses bone formation [28, 29, 35] while enhancing bone resorption [28, 36, 37]
- Sympathetic tone and leptin regulate the expression of molecular clock genes in osteoblasts [29, 32, 38]. Stimulant medications cause sleep disturbances, thereby highlighting another potential mechanism for impaired bone health via SNS stimulation
- Leptin promotes osteoblast-driven bone formation [28, 38] and inhibits osteoclastogenesis [28, 39]. Leptin levels are lower in children with ADHD, with or without methylphenidate treatment [40]. Some [25, 41], but not all [42], studies have shown decreases in leptin with stimulant medications, likely due to loss of fat mass [25]
- Central dopamine signaling (i.e., increased dopamine levels in the brain, as occurs with psychostimulants) is catabolic to bone, likely mediated by $\:{\upbeta\:}$_2_-receptor activation [43, 44]. Conversely, peripheral dopamine signaling through dopamine receptors on osteoblasts and osteoclasts is anabolic to bone, via inhibition of osteoclastogenesis and stimulation of osteoblastogenesis [43, 45].

#### Summary

By increasing norepinephrine levels (directly or through conversion from dopamine), psychostimulants can impair bone health via β_1_- and β_2_-receptor stimulation and possibly decreased leptin levels [28]. Importantly, the SNS-driven inhibition of bone formation appears to be independent of effects on body weight [29, 46]. Therefore, these pathways provide a mechanistic basis for the BMD impairments observed in some studies, independent of the medications’ effects on weight, growth and physical activity.

## Bone Health and Psychostimulant use (Fig. 1)

### Animal Data

Animal studies have demonstrated lower appendicular (but not vertebral) BMD and poorer bone strength in 4-week-old male rats given methylphenidate for 13 weeks compared to controls [47]. The authors suggested these skeletal differences were at least partially independent of methylphenidate’s effects on body weight, despite positive correlations, and could be due to alterations in testosterone [47]. Interestingly, these detriments in skeletal structure and strength were reversed after 5 weeks of non-use [47]. This potential for skeletal recovery is notable because it (1) reinforces the importance of interpreting the literature examining this relationship in past vs. current users, and (2) highlights that long-term skeletal consequences of childhood/adolescent use could be avoided if medication cessation occurs when bone modeling can still recover, similar to growth curve recovery observed after cessation [1].

In a subsequent study, femoral but not vertebral, bone strength was impaired in male, but not female, rats treated with high dose methylphenidate for 13 weeks [48]. Increased bone resorption was evident on histomorphometry in both sexes, although greater in males [48]. Methylphenidate induced osteoclast maturation and activity in cell culture, particularly in those supplemented with male rat serum [48]. Together, these data suggest chronic methylphenidate use impairs bone health more in male than female rats, due to a direct effect on osteoclasts [48].

### Cross-sectional and Longitudinal Comparisons of Bone Turnover and BMD in Children with ADHD

Lahat et al. performed a cross-sectional study that measured bone turnover markers (BTMs; e.g., bone specific alkaline phosphatase [BSAP], urinary deoxypyridinoline [DPD]) and BMD at the lumbar spine (L-spine) and proximal femur using dual photon absorptiometry (QDR-1000) in 10 boys diagnosed with ADHD, treated with methylphenidate for 1–2 years and 10 controls matched for sex, age, height, weight and ethnic origin [49]. No differences were observed between the two groups [49]. However, the marker of bone formation (BSAP) was numerically lower and marker of bone resorption (urinary DPD) numerically higher in the boys on methylphenidate, consistent with the effects of heightened SNS tone. The study may have been underpowered, as data were only available in 9 of 10 participants in each group.

In a prospective study, Poulton and colleagues examined BTMs and body composition and BMD by dual-energy x-ray absorptiometry (DXA) in children (85% male, age: 4.7–9.1 years) with newly diagnosed ADHD, treated with methylphenidate or dexamphetamine [25]. At the 3-year follow-up, BTM and BMD data were available in 14 of the original 34 participants [25]. The bone formation marker, P1NP, was significantly lower after the initial 3 months of medication use but was above baseline levels at 3 years. CTX (bone resorption marker) and osteocalcin (traditionally regarded as a marker of bone formation that reflects some bone resorption) were significantly higher after 3 years [25]. When adjusted for sex, age, and height, total body and L-spine BMD were significantly higher at baseline, 3 months and 3 years in children on stimulant medications compared to controls but importantly, bone accrual was slower than expected for growth in height [25]. Bone mineralization lags behind linear growth, therefore, higher BMD in children using psychostimulants may have been confounded by impaired linear growth. Of note, BMD data for stimulant-treated children and controls were obtained on different machines (GE Lunar Prodigy vs. GE Lunar DPX, respectively) [25]. No differences between methylphenidate and dexamphetamine were observed, though this may have been due to the small sample size [25].

An analysis of data from four observational cohorts of 5 to 17 year olds on risperidone (100% male, mean 11.7 $\:\pm\:$ 2.8 years) with or without concurrent stimulant medication, identified no association between intermittent or continuous psychostimulant use and BMD at the ultradistal radius or L-spine [50]. However, the association may have been masked by the concurrent use of risperidone and SSRIs with subsequent effects on weight and/or partial adherence to stimulant medication that was not captured by medical record review [50].

A retrospective study of children presenting with a distal radius fracture identified 52% lower bone density in children with ADHD taking methylphenidate or mixed amphetamine salts for 1–5 years (*N* = 62) compared to age- and sex-matched ADHD controls who were not on medication (*N* = 126) [51]. Using optical density, no difference in bone density was found between those taking stimulant medications for 5 + years and controls [51].

### Epidemiological Analyses

Three analyses of cross-sectional data from the National Health and Nutrition Examination Survey (NHANES) suggest that current stimulant medication use, particularly for $\:\ge\:$3 months, is associated with lower BMD in children and adolescents [52–54].

- Using 2005–2010 NHANES data, Howard et al. identified lower BMD at the L-spine, femoral neck and total hip in 8–17 year olds on prescription stimulants (*N* = 167, 71.5% male, mean age $\:\pm\:$ SD: 12.18 $\:\pm\:\:$3.23 years) compared to propensity-score matched children and adolescents that were not on any medication [54]. Although the directionality of these findings is consistent with other NHANES analyses, the magnitude of difference may be exaggerated due to lack of adjustment for height Z-score and other relevant confounders [53, 54].
- Feuer et al. also used 2005–2010 NHANES data to examine the association between stimulant medication use and DXA-derived BMD at the L-spine and hip. Compared to Howard et al. [54], Feuer et al. used a broader age range (8 to 20 years) and adjusted for relevant confounders [53]. The 159 individuals who reported stimulant use (71% male, mean age $\:\pm\:$ SD: 12.23 $\:\pm\:\:$2.79 years males, 11.85 $\:\pm\:$ 2.93 years females) had been receiving the medication for 1,120 ± 916 days [53]. Stimulant use was associated with lower BMD at the L-spine, total femur, and femoral neck which persisted after adjustment for age and sex [53]. Stimulant use was still associated with lower L-spine BMD after additionally adjusting for race or ethnicity, height and weight Z-scores, poverty income ratio, physical activity level and cotinine level but was no longer statistically significant for the femoral neck (*p* = 0.08) or total femur (*p* = 0.52) [53]. When stratified by duration of medication use, significant associations between lower BMD at the L-spine and femoral neck were seen in those that had been on medication for $\:\ge\:$3 months but not shorter durations [53].
- Fu et al. examined the relationship between stimulant medication use and DXA-derived BMD at the L-spine, pelvis and total body in 8–16 year olds using 2011–2018 NHANES data [52]. The 284 participants on stimulants (75% male, mean age $\:\pm\:$ SD 11.3 $\:\pm\:$ 2.3 years) reported use for an average of 943.1 ± 828.2 days [52]. BMD was lower at the L-spine, pelvis and total body in children and adolescents on stimulant medications compared to nonusers [52]. In subgroup analyses, these associations remained statistically significant for males and $\:\ge\:$3 months of use. However, the subgroup analyses in women and those on stimulant medications for $\:\le\:$3 months may have been underpowered.

It is unclear what, if any, proportion of the “nonuser” comparator groups in these NHANES analyses had untreated ADHD. It is also unclear if lower BMD in ADHD-treated children and adolescents is mediated by behavioral modifications that decrease physical activity

One study examined ADHD medication use and BMD in adults using 2013–2018 NHANES data for 18–50 year olds [55]. The 90 participants (60% male, avg ~ 34 years) had been taking ADHD medication for an average of 504 $\:\pm\:$ 663 days. Adults on ADHD medication had lower BMD at the skull and thoracic spine compared to nonusers. Of note, ADHD medication use included stimulants and non-stimulants. Therefore, the analysis of stimulant medications and BMD may have been underpowered, particularly given the limited sample size. In addition, BMD data were derived from a body composition DXA scan, rather than the gold-standard central DXA.

### Summary (Fig. 1)

Animal data [47, 48] support a mechanistic link between current stimulant medication use and lower bone density/strength at appendicular sites [47] due to direct effects on osteoclasts, particularly in males [48]. Human data suggest psychostimulants slow the rate of bone mass accrual in childhood [25]. Furthermore, $\:\ge\:$3 months of psychostimulant use is associated with lower BMD in childhood through young adulthood in epidemiological studies [52–54]. These studies used predominantly male children that had been using medication for $\:\ge\:$1 year. ADHD prevalence is higher in males during childhood [2] but sex differences diminish with age and is nearly equal in population surveys [2]. In addition, no studies have assessed if/how these medications affect acquisition of peak bone mass or stability of BMD in individuals who remain on medication through mid/late-life. Therefore, more research is needed on medication use in adulthood, particularly women. Data from a recently completed 1-year observational study that examined BMD accrual during puberty using DXA and High-Resolution peripheral Quantitative Computed Tomography (HR-pQCT) in children with ADHD initiating stimulant medication will address some of these knowledge gaps (R01 HD101326, PI Calarge). Since BMD is only one determinant of fracture risk, it is important to understand how these medications contribute to fracture risk, independent of their detrimental effects on bone metabolism and BMD.

**Fig. 1:**
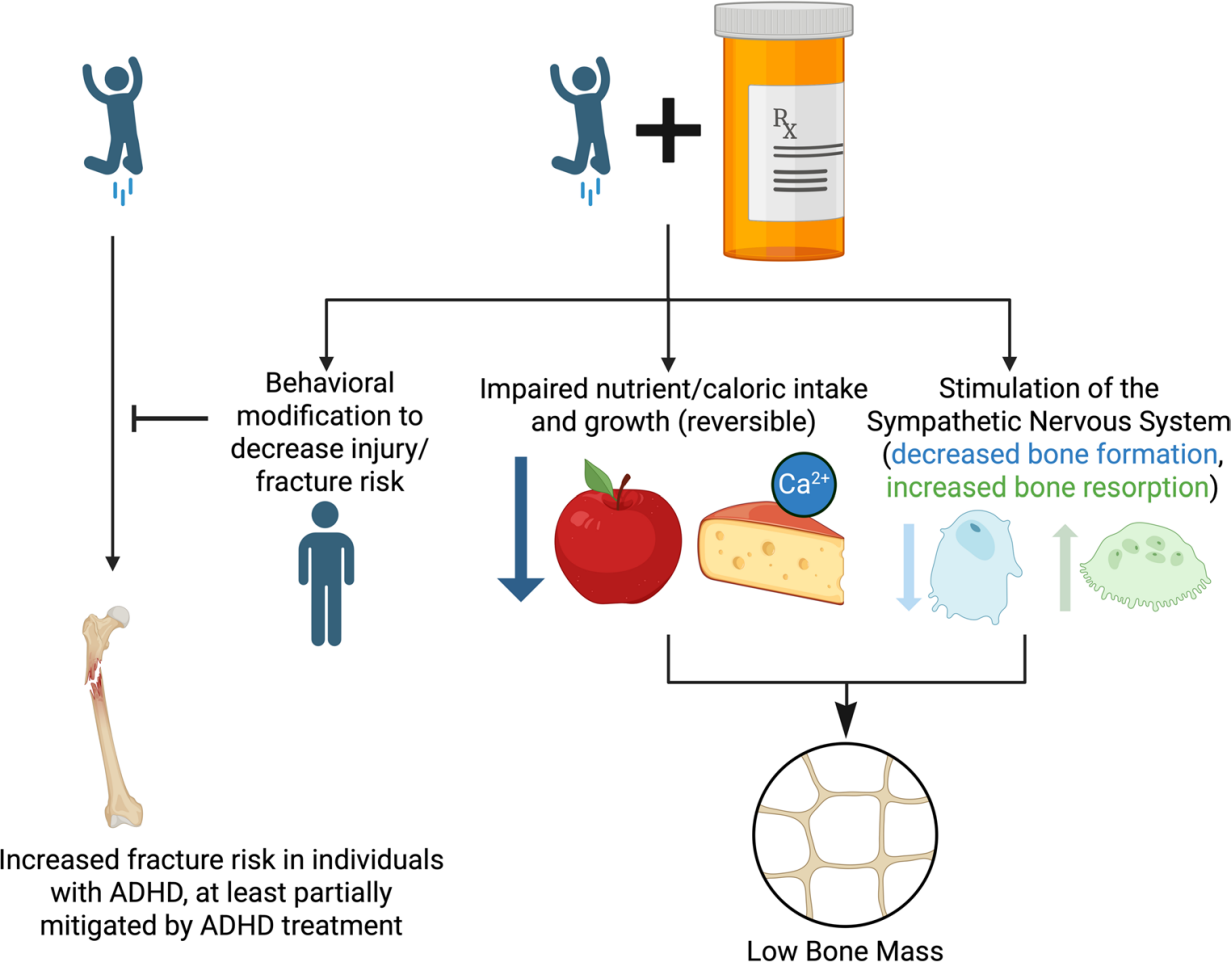
Proposed framework for the relationship between stimulant medications and bone health

Individuals with Attention Deficit/Hyperactivity Disorder (ADHD) have an increased risk of injury, including fracture. Most data suggest that current use of stimulant medications for ADHD decrease the risk of injury and traumatic fracture due to behavioral modifications. Stimulant medications may cause lower-than-expected peak bone mass during childhood/adolescence due to impaired nutrient/calorie intake, including calcium-containing foods, with impaired height/weight growth trajectories, and/or through stimulation of the sympathetic nervous system (SNS), which regulates bone metabolism. Created in BioRender. Swanson, C. (2026) https://BioRender.com/nf86c7z.

## Stimulant Medication use May Mitigate the Higher Fracture Risk in ADHD and Influence Fracture Healing

Children with ADHD are at higher risk for injury compared to children without ADHD [56, 57]. Data suggest this increased risk is partly related to ADHD behavioral manifestations, persists into adulthood [57, 58], and decreases with medication treatment [4, 56, 59–61]. 

Although estimates vary, ADHD approximately doubles the risk of fracture in both sexes from childhood through at least 25 years old [28, 57, 62–73], with some [58] but not all [70] data suggesting the increased fracture risk extends into adulthood. Two articles that utilized the same data from the Longitudinal Health Database in Taiwan from 2000 to 2009 reported that ADHD increases fracture risk more in girls than boys [63, 64]. The increased fracture risk observed in young patients with ADHD spans different types of fracture (e.g., traumatic, stress), involves similar anatomical locations as fractures in children without ADHD (upper limbs > lower limbs > skull/face/trunk) [28, 63, 64, 74], and is thought to be related, in part, to impulsivity, inattention and hyperactivity that result in increased risk of accidental injury [28, 62, 71, 74, 75]. 

Consistent with this hypothesis, most studies have demonstrated a decrease in fracture risk [68, 73, 74] in children, adolescents [28, 64, 76, 77] and young adults [62, 75] with ADHD treated with stimulant [22, 70] and sometimes non-stimulant medication. Importantly, these analyses focused on medication use at the time of the fracture event or in the months preceding it. The fracture risk reduction observed with psychostimulant medication may be greatest in younger populations ($\:\le\:$25 years old) [76]. Fracture risk reduction observed in individuals with ADHD treated with either stimulant and non-stimulant medication [74] reinforces the hypothesis that increased fracture risk in ADHD is due, at least in part, to the behavioral manifestations and that any treatment that effectively modifies behavior (e.g., reduced impulsivity) is beneficial. However, not all studies demonstrate fracture risk reduction with non-stimulant medications [70]. Therefore, studies reporting reduced fracture risk with stimulant medications may be biased by higher prevalence of use or confounded by more severe symptomatology [74], thereby over-estimating the risk reduction in patients that use psychostimulants for mild or no ADHD symptoms.

When duration of medication use is considered, fracture risk reduction is most consistently observed with longer use. For example, longer duration of stimulant medication use (defined as $\:\ge\:$2 prescriptions or >180 days) was associated with fracture risk reduction [62, 74–77], with inconsistent sex differences. One publication observed reduced fracture risk with shorter duration of use but also noted progressive reductions with longer durations in men [62]. It is unclear how this association changes over decades of use and the maximum duration of use associated with fracture risk reduction is not known.

The association between stimulant medications and fracture risk reduction in individuals with ADHD may be driven by *traumatic* fractures [78], as the data regarding stress fracture risk are more mixed. Some publications have found a small *increased* risk of stress fracture with past [69, 79] use of methylphenidate while others show reduced risk with current use [22], particularly longer duration [75]. Four studies utilized medication and fracture data from the predominantly male Israeli Defense Forces [62, 69, 75, 79]. Only one of these found that the medication-induced fracture risk reduction was sex-dependent, with decreased risk in men currently on methylphenidate, but not women [62]. 

Similar to data in ADHD, one study identified higher fracture risk in adults with narcolepsy [80]. Stimulant medication use was associated with a lower incidence of fractures compared to non-users, although this was not statistically significant after accounting for relevant confounders [80]. 

### Stimulant Medication use and Fracture Healing

Use of methylphenidate or dextroamphetamine for $\:\le\:$5 years prior to distal radius fracture was found to impair bone healing (as assessed by optical density) in 6–18 year olds with ADHD compared to age and sex-matched nonuser ADHD controls [51]. In this predominantly male cohort, radiographic healing by optical density was observed after 6 weeks in those on medications compared to 4 weeks in controls [51]. Based on preclinical evidence from the same group on the effects of methylphenidate on bone and osteoclasts [47, 48], the authors speculated that the delayed fracture healing in this retrospective analysis was due to impaired bone remodeling which interfered with the final phase of fracture repair [51].

### Summary

Most data demonstrate that current stimulant use is associated with decreased fracture risk in children/adolescents with ADHD. These mostly retrospective analyses rely on electronic medical record coding to identify the exposure (prescription and/or pharmacy records used as a surrogate for medication use) and outcomes (fracture incidence). Although many have excellent follow-up duration and observed relatively healthy cohorts [62] with subsequently less potential confounding due to other comorbidities or medications, there are inherent limitations to retrospective database analyses [28] that make causation and/or mechanistic determination challenging. Furthermore, the literature has primarily focused on child/adolescent populations. The skeletal implications of prolonged stimulant medication use, either during growth and/or continuing throughout adulthood, and in older adults already at increased risk for fracture, have not been evaluated. Importantly, data that indicate lower fracture risk in patients with ADHD treated with stimulant medications cannot be extrapolated to populations that use stimulant medications off-label or illicitly. If the ultimate effect of stimulant medications on bone health is a balance between detrimental effects on bone metabolism and beneficial behavioral modification, the risk/benefit ratio may differ in those that do not use the medication to control moderate to severe ADHD behaviors.

## Strengths and Limitations

This narrative review has many strengths, including a comprehensive review of relevant literature, inclusion and synthesis of studies with diverse experimental designs and occasionally contradictory findings, and a critical appraisal of the available literature. The manuscript also has some limitations. For example, a more limited scope may have provided a more focused, concise review. However, by evaluating the current body of literature across the lifespan, the review helps to identify areas in need of further investigation.

## Conclusions

BMD is often, but not always, lower in children, adolescents and adults that use stimulant medication for ADHD. This is likely mediated, at least in part, by the medications’ potentially reversible effects on weight, nutrition, and decreased physical activity, and SNS stimulation that limits bone formation but not resorption. Despite apparent detrimental effects on bone metabolism/density, current stimulant medication use reduces fracture risk in most studies, when used in children/adolescents with ADHD for at least 6 months, likely due to behavioral modification. Age of medication cessation may be an important factor. After skeletal maturity, the deleterious effects of psychostimulants on bone mass may be irreversible, while the underlying behavioral risks of injury persist. Therefore, the ultimate effect of stimulant medications on bone health may be a balance between effective behavioral modification and negative skeletal effects mediated by SNS stimulation and nutrition. Age, sex, duration of medication use, potential for BMD recovery after or with periods of intermittent cessation, and the presence/severity of underlying ADHD symptomatology may influence this delicate balance and ultimately increase fracture risk in some. This may be important to consider when the generations that experienced expanded use of psychostimulants mature and are screened for or diagnosed with osteoporosis.

Importantly, given the known benefits of ADHD treatment [2], appropriate evaluation, diagnosis and treatment are critical. A comprehensive treatment approach, including behavioral therapy, may allow for intermittent psychostimulant cessation and/or use of lower doses, thereby potentially decreasing the risks of excess sympathetic tone on bone. Until more is known about how psychostimulants affect fracture risk across the lifespan, it is prudent to counsel patients and their families on lifestyle measures to optimize bone health including, but not limited to, optimal calcium and vitamin D intake, physical activity, smoking avoidance/cessation, and fall prevention.

Future research should investigate how psychostimulants affect acquisition of peak bone mass, stability of bone mineral density (BMD) when used through mid/late-life, particularly in women, and fracture healing.

## Data Availability

No datasets were generated or analysed during the current study.
